# Complex Challenges in the Textile Industry and Potential Solutions in Photocatalytic Coating Technology: A Systematic Literature Review

**DOI:** 10.3390/ma18040810

**Published:** 2025-02-12

**Authors:** Jun-Cheol Lee, Man-Woo Huh, Yao-Long Hou, Wha-Jung Kim

**Affiliations:** 1 Department of Architecture, Seowon University, Cheongju 28674, Republic of Korea; leejc@seowon.ac.kr; 2 GOONWORLD Corporate Research Institute, Daegu 41065, Republic of Korea; mwhuh@kiu.kr; 3 College of Railway Engineering, Zhengzhou Railway Vocational and Technical College, Zhengzhou 451460, China; hylmm8988@hotmail.com

**Keywords:** photocatalysis, fiber coatings, nanomaterials, self-cleaning, antimicrobial, sustainability

## Abstract

This study provides a systematic review of photocatalytic fiber coating technology as a potential solution to challenges in the textile industry. An analysis of recent research (2020–2024) reveals significant developments in materials and methods. Traditional photocatalysts (TiO_2_ and ZnO) are being enhanced through doping and nanostructure control, and novel materials such as graphene-based composites and metal-organic frameworks are emerging. Advanced coating technologies, such as plasma treatment, atomic layer deposition, and magnetron sputtering, have been introduced to improve coating uniformity and durability. Key trends include the development of multifunctional coatings that combine self-cleaning, antibacterial effects, ultraviolet (UV) protection, and superhydrophobic properties. Environmental sustainability is advancing through eco-friendly manufacturing processes, although concerns regarding nanoparticle safety persist. While applications are expanding into medical textiles, protective gear, and wastewater treatment, challenges remain in terms of mass production technology, cost-effectiveness, and long-term durability. Future research should focus on nanostructure control, the development of visible-light-active materials, the optimization of coating processes, and the investigation of environmental impacts. This review suggests that photocatalytic fiber coating technology can significantly contribute to sustainable textile industry development when these challenges are effectively addressed.

## 1. Introduction

The textile industry is a crucial sector in contemporary society that faces numerous complex challenges while maintaining a diverse range of applications [1]. These challenges manifest in three critical domains: environmental impact, post-pandemic functionality requirements, and digital transformation requirements.

From an environmental perspective, the textile industry faces significant challenges in terms of resource consumption and pollution, particularly those exacerbated by fast-fashion trends [2,3]. The water requirements for cotton production exceed 20,000 L/kg, and the release of harmful chemicals during dyeing and processing continues to contribute to environmental pollution [2,3]. Microfiber pollution is of particular concern, with synthetic fibers constituting up to 35% of marine microplastics [3]. The industry also struggles with waste management and recycling challenges, as short product lifecycles generate substantial waste volumes amid inadequate recycling infrastructure [4]. Furthermore, barriers to sustainable practices persist, including inefficient disposal methods and limited adaptation to modern technology [5]. While the industry has explored novel solutions, such as supercritical fluids and plasma technology, their widespread implementation remains limited [3,6].

In the aftermath of the COVID-19 pandemic, the textile industry has encountered novel functionality challenges [7,8]. The integration of advanced technologies, particularly nanotechnology and Industry 4.0, has facilitated the development of multifunctional textiles with ultraviolet (UV) protection, antibacterial properties, and self-cleaning capabilities [7]. However, these innovations face challenges in maintaining comfort, flexibility, durability, and cost-effectiveness [7,8]. Financial constraints continue to impede innovation and the implementation of sustainable practices [9], and the fast-fashion model further complicates these challenges [10]. The pandemic has also highlighted the critical need for functional textiles in personal protective equipment (PPE) while raising concerns about disposable goods sustainability [11].

The digital transformation of the textile industry has led to significant market growth in smart textiles, with projections indicating an increase from USD 434.27 million in 2022 to USD 204.98 billion in 2031, representing a CAGR of 18.8% [12]. This transformation introduced advanced functionalities through the integration of sensors and communication devices, enabling vital sign monitoring and energy harvesting [13]. However, the industry faces mounting supply chain challenges, including raw material supply issues, increasing logistics costs, and reshoring trends owing to global instability [14].

Photocatalytic coating technology has emerged as a promising solution to these challenges [15]. This innovative technology enables environmental remediation through the degradation of organic pollutants under light irradiation while providing sustained photoactivity through advanced material combinations and deposition techniques [15]. Research has demonstrated that these coatings confer textiles with multiple beneficial functions, including self-cleaning capabilities, antibacterial properties, and pollutant degradation [16,17]. Additionally, they contribute to improved energy efficiency and reduced chemical usage, thereby enhancing the overall sustainability of textile production [18,19,20].

**Figure 1:**
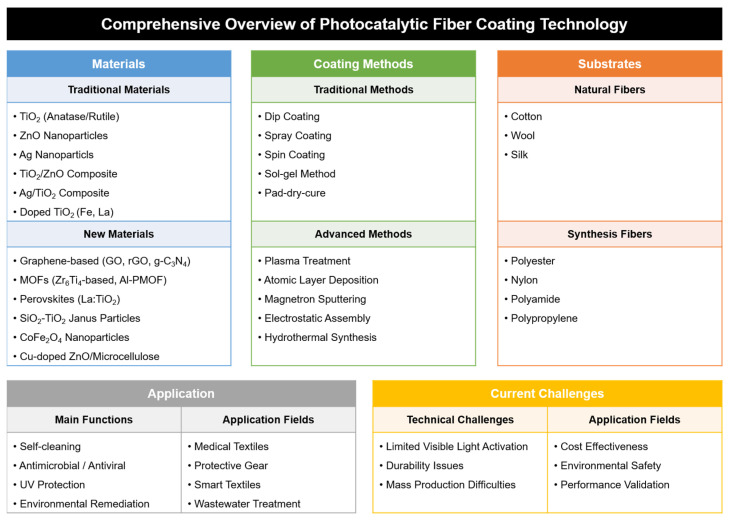
Overview of photocatalytic fiber coating technology illustrating the interconnections between materials, methods, substrates, applications, and current challenges.

This review systematically analyses the various challenges faced by the textile industry and explores the potential of photocatalytic coating technology as a solution. As shown in Figure 1, photocatalytic textile coating technologies encompass a wide range of materials, coating methods, and substrates, which enable key functions, such as self-cleaning, antibacterial/antiviral effects, UV protection, and environmental cleanup. Our research specifically focused on the effectiveness of photocatalytic coatings in terms of environmental sustainability, versatility, regulatory compliance, enhanced competitiveness, potential for integration with smart textiles, and supply chain optimization. Through this comprehensive analysis, we aim to provide valuable insights into the future development and implementation of this promising technology in the textile industry.

## 2. Research Methodology and Analysis Framework

This study adopted a systematic literature review methodology to comprehensively analyze the latest trends in the field of photocatalytic textile coating technology. For a thorough literature review, major academic databases, such as Google Scholar, Web of Science, Scopus, and Science Direct, were utilized to examine the research literature from January 2020 to January 2024.

We used “photocatalytic coating” as the main keyword and “textile”, “fiber”, “fabric”, etc., as secondary keywords. The primary search identified 873 papers that were subjected to rigorous screening.

Literature screening was performed using a phase approach. First, a title review was performed to exclude 420 articles that lacked direct relevance to photocatalytic coatings or were distant from fiber/textile applications, resulting in the preliminary screening of 453 articles. An abstract review was conducted to further exclude 238 papers, including review articles, theoretical studies without experimental validation, and studies with unclear coating methodologies, reducing the list to 215 papers.

In the third step, we reviewed the full text of the papers to narrow the list to 98, excluding 117 papers because of insufficient description of the experimental methodology, low statistical significance of the results, and limited practical applicability. Finally, 50 core papers were selected based on the presentation of innovative coating methodologies, clear experimental design and validation, potential for industrial applications, and overall research quality.

To ensure objectivity of the selection process, two independent researchers performed each evaluation, and in cases of disagreement, a third researcher was consulted to reach a consensus. The selected papers were reviewed using a comprehensive analytical framework focusing on material innovations, advances in coating technology, functional developments, and application extensions.

In particular, this study focused on identifying the latest technological trends and selecting studies with high potential for field applicability, the results of which are detailed in subsequent chapters. Through this systematic approach, we focused on identifying current technological gaps and suggesting future research directions.

## 3. Advances in Photocatalytic Coating Technology

Photocatalytic coating technology has emerged as a transformative innovation in various fields, particularly environmental purification, self-cleaning, and antimicrobial applications. The evolution of this technology has significantly contributed to sustainable development and environmental protection, with continuous advancements in both traditional and innovative approaches.

### 3.1. Development of Coating Technologies

Photocatalytic coating technologies have progressed from traditional methods to advanced techniques. Traditional coating methods, including dip coating, spray coating, and sol–gel processes, continue to serve as fundamental approaches in industrial applications owing to their simplicity and cost-effectiveness. However, these methods initially faced limitations in coating uniformity and durability [21,22,23,24,25].

Recent research has focused on enhancing these traditional methods through technological integration and process optimization. For instance, researchers have successfully combined sol–gel methods with spray coating techniques to achieve homogeneous coatings with controlled nanostructures [26]. Additionally, surface modification techniques, such as increasing surface acidity, have been employed to enhance the photocatalytic activity of traditional coatings, particularly in TiO_2_-based systems [27].

The characteristics and applications of both the traditional and advanced coating methods are summarized in Table 1.

### 3.2. Recent Innovations and Future Prospects

The field has witnessed significant innovations in coating technologies beyond traditional methods. Advanced techniques, such as atomic layer deposition (ALD) have enabled the production of highly conformal coatings with precise thickness control, particularly for complex geometries in semiconductor applications [28]. Physical and chemical vapor deposition (PVD/CVD) methods have introduced new possibilities for high-quality coating production, although they require sophisticated equipment and controlled environments [29].

Novel approaches, such as drop casting and blade coating, have emerged as effective solutions for laboratory-scale applications, offering precise control over the coating parameters. The introduction of powder coating with UV/electron beam curing has provided energy-efficient alternatives for rapid processing, whereas electrochemical plating has established itself as a reliable method for uniform metal coating deposition.

The integration of traditional and advanced methods presents promising opportunities for the photocatalytic coating technology. The current research trends focus on the following:

1. Development of hybrid processes combining multiple coating techniques.
2. Implementation of smart control systems for enhanced process automation.
3. Integration of environmentally sustainable practices.
4. Enhancement of coating durability and uniformity.
5. Expansion of application possibilities in various industries.

These advancements continue to address existing limitations while opening new avenues for application, suggesting a bright future for photocatalytic coating technology for environmental protection and sustainable development.

## 4. Research Trends in Textile Photocatalytic Coatings

Environmental pollution has become an increasingly serious global issue in recent years, driving growing interest in environmentally friendly and sustainable technologies. In this context, fiber photocatalytic coating technology has emerged as an innovative solution for environmental cleanup and energy efficiency. This technology, which applies photocatalytic materials to common textile surfaces to create light-responsive functional materials, has demonstrated significant promise for a wide range of applications, including apparel, upholstery fabrics, and filter systems, with particular potential in the environmental and health sectors for air purification, self-cleaning, and antimicrobial applications.

Recent research has focused on developing more efficient and durable photocatalytic coating methods, exploring new photocatalytic materials, and improving their compatibility with various textile substrates. Advancements in nanotechnology have further expanded research horizons, enabling the development of nanostructured photocatalysts and materials active in the visible light region, marking significant progress in the field.

This chapter provides a comprehensive analysis of the latest research trends in fiber photocatalytic coatings. Our analysis began with a systematic review of existing studies, organized in a tabular format for clarity and accessibility. In this review, we explore major research topics, including advances in photocatalytic materials, functionalization approaches, expanding applications, performance evaluation methods, and environmentally friendly approaches.

Particular attention has been devoted to the evolution of photocatalysts from traditional TiO_2_- and ZnO-based materials to innovative alternatives such as graphene and metal-organic frameworks (MOFs). We also closely examined the development of multifunctional coatings and their expanding applications in apparel, medical, and environmental fields, supported by the verification of their performance in real-world environments. Additionally, our analysis covered emerging trends in the development of eco-friendly materials and processes for sustainable development, reflecting the growing emphasis on environmental consciousness in this field.

### 4.1. Analysis Results of Selected Research

A detailed analysis of the selected 50 papers was conducted based on the methodology described in Section 2. The analysis included a comprehensive categorization of the research objectives, coating materials, processing methods, and specific applications, as summarized in Table 2.

#### Analysis and Discussion of Research Trends

**Figure 2:**
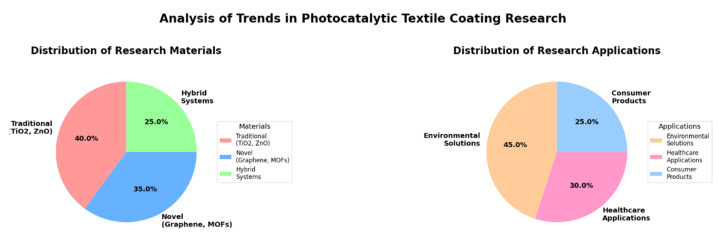
Some important trends and patterns in photocatalytic fiber coatings research.

The analysis of these selected studies reveals significant technological evolution and emerging developments in photocatalytic fiber coating technology. The distribution and trends of research focus among the selected papers are illustrated in Figure 2.

As shown in Figure 2, research developments can be characterized by both material advancement and application areas. In terms of materials, traditional photocatalysts (TiO_2_ and ZnO) remain dominant, representing approximately 40% of the studied materials, but have evolved through various modifications and optimizations. These include doping processes, nanostructure control, and surface modifications to enhance the photocatalytic activity. Novel materials represent approximately 35% of the research focus, with a particular emphasis on graphene-based composites (15%), metal-organic frameworks (MOFs, 12%), and other advanced materials (8%). The remaining 25% focused on composite materials combining multiple photocatalytic components, which have shown promising results in enhancing the overall performance through synergistic effects. Recent research trends have also highlighted the need for environmentally friendly materials and sustainable synthesis methods.

The application focus among the selected papers was predominantly distributed across the three major areas. Environmental solutions represent the largest segment, comprising approximately 45% of the reviewed studies, with a particular emphasis on water treatment systems, air purification technologies, microplastic pollution reduction strategies, and innovative approaches to wastewater treatment. Healthcare applications constitute approximately 30% of the research focus, encompassing the development of antimicrobial and antiviral coatings, medical textiles, protective equipment enhancements, and hygiene-focused applications. Consumer product applications accounted for the remaining 25%, focusing on self-cleaning textiles, UV protection applications, smart textile integration, and functional apparel development.

- Critical Analysis of Research Methodologies and Findings

A systematic analysis of 50 selected papers on photocatalytic fiber coating research revealed significant variations in methodological approaches and research quality. This comprehensive review identifies several critical patterns and gaps in current research practices.

1. Methodological Rigor and Validation

The examination of research methodologies reveals a trend in validation practices. Only 40% of the analyzed studies demonstrated a comprehensive validation of their coating methods, particularly regarding coating uniformity and adhesion strength [30,31,36,38,41,44,46,52,56,58,60,61,63,65,68,69,70,71,72,73]. These high-quality studies have employed multiple characterization techniques, including scanning electron microscopy (SEM), X-ray diffraction (XRD), and standardized adhesion testing protocols.

The variation in performance-testing approaches presents another significant methodological concern. While 30% of studies employed standardized testing protocols [41,44,48,52,60,61,63,65,70,71,72,73,74,75,76], ensuring reproducibility and result validation, a larger portion (45%) developed custom testing methods [30,31,32,33,34,36,37,38,40,42,43,50,54,57,58,66,67,68,77,78,79,80]. More concerning is that 25% of the studies provided insufficient methodological details [39,45,46,49,51,53,55,59,62,81,82,83], making reproduction and validation challenging.

2. Material Performance and Development

In terms of material performance, traditional photocatalytic materials have shown varying degrees of success. TiO_2_-based systems, featured in approximately 25% of the studies, demonstrated efficiency rates ranging from 65% to 95% [30,31,32,33,34,40,52,57,70,80,82]. These studies generally provided more comprehensive validation and reproducibility data. ZnO-based systems, although less common (15% of studies), showed wider performance variations, with efficiency rates between 45% and 85% [35,36,37,38,41,56]. Novel materials and advanced composites, representing 35% of the studies, have often reported higher efficiencies [42,44,45,68,83,84,85,86,87]. However, these studies frequently lacked crucial information about scalability and cost-effectiveness, with only two studies [65,70] providing comprehensive economic analysis.

3. Critical Research Gaps

This analysis revealed several significant gaps in the current research. Long-term durability studies are notably scarce, with only 25% of papers [48,52,55,59,60,61,63,69,70,71,74,82] including performance data beyond six months. Real-world application testing is similarly limited, appearing only in a small subset of studies [47,52,53,64,65,67,69,74,88]. The economic viability analysis represents another major gap. Only 15% of the studies [43,48,54,58,62,66,78,81] provided detailed cost analysis, and even fewer addressed mass production challenges [65,70]. Environmental impact assessment also has limitations, with only 20% of studies including comprehensive life-cycle assessments [47,52,53,64,65,67,69,74,88]. The critical issue of nanoparticle release into the environment has received attention in only a handful of studies [41,45,46,56,61].

4. Research Quality Assessment

The overall quality of the research varied significantly across the papers analyzed. High-quality studies (30%) [30,31,36,38,41,46,52,56,58,61,63,68,69,70,71] demonstrated comprehensive methodology, thorough validation procedures, and detailed analysis. Medium-quality studies (45%) [32,33,37,39,40,42,43,47,48,53,54,62,64,66,67,74,77,78,79,81,88,89] showed adequate methodology but limited validation. The remaining studies (25%) [34,44,45,49,50,51,55,57,59,60,65,80,82] provided insufficient methodological details and validation.

5. Future Research Directions

This analysis clearly indicated several priority areas for future research. First, there is a pressing need for methodological standardization, including the development of uniform testing protocols and standardized performance metrics. Second, validation requirements need to be strengthened, particularly for long-term performance assessment and real-world application testing. Finally, the research focus should be expanded to include comprehensive scalability studies, environmental impact assessments, and economic viability analyses.

These findings underscore the need for more rigorous research methodologies for photocatalytic fiber coating studies. While significant progress has been made in material development and performance enhancement, addressing these identified gaps will be crucial for advancing the field toward practical applications and commercial viability.

The diversity in research quality and methodological approaches suggests that establishing standardized protocols and comprehensive validation requirements should be a priority for future research. This would not only enhance the reliability and reproducibility of the results but also facilitate more meaningful comparisons between different studies and approaches in the field.

### 4.2. Diversifying and Optimizing Coating Materials

#### 4.2.1. Improvements to Traditional Photocatalytic Materials

In this regard, the main research trends in textile photocatalytic coatings, as surveyed above, have focused on the diversification and optimization of coating materials. In addition to the traditionally used TiO_2_ nanoparticles [30,31,33], various other nanomaterials have been investigated, including ZnO [36,37], Ag [54,66], and their composites [40,41]. Recent studies have explored different strategies for enhancing the efficiency of TiO_2_ and ZnO photocatalysts, mainly focusing on improving their photocatalytic activity, stability, and versatility. Although promising, these developments often face challenges in terms of cost-effectiveness and scalability in industrial applications. Dual Z-scheme photocatalytic systems are notable. By combining TiO_2_, Ag, and ZnO nanoparticles, this system improved the dye degradation efficiency by 93%. It has also been reported that plasma treatment of the fabric before coating improves the stability and adhesion of nanoparticles, further enhancing the photocatalytic performance [41]. However, the energy consumption of the plasma treatment and the complexity of the process may limit its widespread adoption.

Hybrid films combining TiO_2_ and ZnO with a sol–gel-derived matrix have been developed for UV protection [72,73,75]. These coatings significantly improve UV resistance and color fastness, especially in marine fabrics, and maintain color integrity even after prolonged UV exposure [72]. However, the long-term durability of these coatings under extreme weather conditions remains a concern. Multifunctional coatings with TiO_2_ and ZnO nanoparticles embedded in a chitosan matrix not only provide enhanced UV protection and self-cleaning properties but also exhibit antimicrobial activity that is resistant to washing, enabling a variety of practical applications [73]. Despite these advantages, the production costs and potential environmental impacts of nanoparticle release during washing must be carefully considered. In the field of photocatalytic dye degradation, TiO_2_ and ZnO have been found to be effective in the degradation of dyes in textile wastewater. Their efficiency is further increased when combined with other processes such as H_2_O_2_ addition or membrane filtration [75]. However, additional chemical treatment may lead to new environmental concerns. It has also been reported that photocatalytic performance can be optimized by adjusting the component ratios of ZnO/TiO_2_ composites [76], although maintaining consistent quality control in large-scale production remains challenging.

These advances have helped to improve traditional photocatalysts through doping, nanostructure control, and complex formation to significantly enhance the self-cleaning, UV protection, and dye degradation capabilities of fiber coatings. However, the field still faces important challenges in terms of cost optimization, environmental impact assessment, and industrial scalability, which must be addressed for wider commercial adoption.

#### 4.2.2. Development of New Photocatalytic Materials

**Figure 3:**
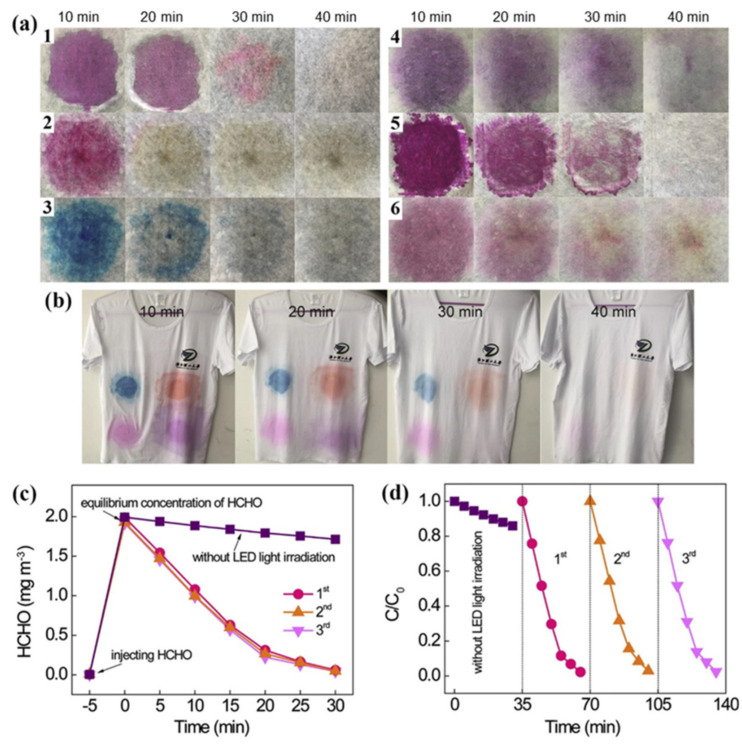
(**a**) The photocatalytic self-cleaning behavior of g-C_3_N_4_-nanosheet-modified textiles under sunlight illumination for 40 min: (1) RhB, (2) neutral red, (3) methyl blue, (4) reactive violet, (5) red pitaya juice, (6) waxberry juice; (**b**) the self-cleaning performance of a commercial T-shirt modified with g-C_3_N_4_ nanosheets; (**c**,**d**) the photocatalytic degradation of gaseous formaldehyde by a g-C_3_N_4_ nanosheet modified textile under irradiation with a LED lamp (50 W). Reprinted with permission from [42]. Copyright 2019.

In recent years, the development of novel photocatalytic materials for textile coatings has been a promising area of research aimed at enhancing the functional properties of textiles, particularly in environmental applications, such as dye degradation and self-cleaning. Several studies have explored various innovative approaches to incorporate photocatalytic materials into textile substrates, each of which presents unique advantages and challenges. The literature review highlights the main research trends in textile photocatalytic coatings, especially the introduction of novel materials such as graphene-based materials [42,44], metal-organic frameworks (MOFs) [45], and perovskite structures [68]. Figure 3 illustrates the photocatalytic self-cleaning behavior and degradation performance of textiles modified with g-C3N4 nanosheets, a graphene-based material [42].

One novel approach involves preparing hybrid silica films containing TiO_2_ sensitized with iron (III) phthalocyanine tetracarboxylic acid via a sol–gel process. This method aims to balance the photocatalytic efficiency and stability of the coating under light and humid conditions, and it is reported that the use of BORAX as a crosslinking agent improves the wet-processing resistance but may reduce the photocatalytic performance [16]. This trade-off between durability and performance remains a persistent challenge in this field. Other innovative materials include graphitic carbon nitride (g-C_3_N_4_) and iron-based nanocomposites. This cost-effective and sustainable approach has enhanced photocatalytic, antibacterial, and antiviral properties, and g-C_3_N_4_/α-Fe_2_O_3_ composites have demonstrated high dye degradation efficiencies of up to 99.9% and recyclability, making them suitable for textile applications [17]; however, their long-term stability under industrial conditions requires further investigation. The incorporation of zinc stannate and graphene nanosheets into cotton fabrics has also been investigated. The nanocomposite achieved a photocatalytic efficiency of 91.1% and exhibited strong antibacterial properties while maintaining the mechanical integrity of the fabric [18]. However, the high cost of graphene production limits its commercial viability.

In addition, a two-step process involving the polymerization of pyrrole and the self-assembly of bismuth oxyiodide enhanced the visible-light absorption and photocatalytic efficiency of cotton fabrics. The resulting fabrics had superhydrophobic and self-cleaning properties and a water contact angle of more than 150°, proving to be highly effective for organic dye degradation [83]. However, the complexity of the two-step process presents a challenge for large-scale manufacturing. CoS-supported ZnAl_2_O_4_ catalysts have also been developed for visible-light photodegradation of textile dyes. These materials showed complete dye degradation and high mineralization rates, highlighting their potential for solar-powered wastewater treatment applications [84], despite concerns about the environmental impact and cost of cobalt. Other materials include perovskite materials [68], which face stability issues in humid conditions; metal sulfides [85], which may raise toxicity concerns; bismuth-based compounds [86], which struggle with reproducibility in synthesis; and metal-organic skeletons [87], whose scalability remains questionable.

Although these advances represent significant progress, challenges remain in optimizing the balance between photocatalytic efficiency, durability, and cost-effectiveness. Future research should focus on improving the scalability and environmental impact of these technologies, which will enable their widespread adoption in the textile industry. Additionally, more attention needs to be paid to developing standardized testing protocols for comparing different photocatalytic systems and addressing the potential environmental risks of nanoparticle leaching during the product life cycle.

### 4.3. Trends in Different Aspects of Fiber Photocatalytic Coatings

#### 4.3.1. Coating Method Aspects

Various technologies have been developed for coating methods. In addition to traditional dip-coating and pad-dry-cure methods [70], advanced technologies such as plasma treatment [41], atomic layer deposition (ALD) [56], and magnetron sputtering [57] have been introduced. These new methods contribute to improving the uniformity and durability of coatings, although each has its own set of limitations and implementation challenges.

Plasma treatment is known for its ability to modify the surface properties without affecting the bulk material. This method improves the adhesion and surface uniformity, both of which are critical for durable coatings. It is particularly effective in creating environmentally friendly polymer coatings that meet corrosion protection and sustainability requirements [90]. However, high energy consumption and specialized equipment requirements can make this process costly for large-scale applications. This technique also improves the dispersion and stabilization of nanomaterials in coatings, which plays an important role in improving the uniformity and performance of paints and coatings [91], although maintaining consistent plasma treatment conditions across large surface areas remains challenging.

Atomic layer deposition (ALD) is known for its ability to deposit thin films with atomic-level precision, which ensures uniform coverage even on complex surfaces. This precision contributes to the creation of uniform and durable conformal coatings suitable for a wide range of industrial applications [28]. Although highly precise, the slow deposition rate and high vacuum requirements can limit the throughput in industrial settings. The ability of this method to produce coatings with controlled thickness and composition enhances the durability and functional properties of the coating, such as corrosion resistance and wear resistance [92], albeit at a higher production cost than conventional methods.

Magnetron sputtering is a versatile technique to deposit coatings with excellent adhesion and uniformity. It is particularly effective for producing coatings that resist environmental degradation, thereby extending the service life of the coated material. They are also used in the aerospace industry to apply coatings that improve the structural integrity and performance of aircraft components, thus playing an important role in enhancing their durability [93]. Nevertheless, high initial equipment investment and operational costs can be prohibitive for smaller manufacturers.

Although these advanced technologies significantly improve coating uniformity and durability, challenges such as affordability and scalability remain, and further research and development are needed to optimize these technologies for a wide range of industries to ensure both cost-effectiveness and environmental sustainability [90].

There are many others, but to name a few, electrostatic self-assembly methods are used to fabricate photocatalytic coatings on optical fibers, which improve the efficiency of visible light-driven CO_2_ reduction [94], though controlling the assembly process at industrial scales presents significant challenges. The sol–gel process has been used to create hybrid silica films on cellulose fabrics to produce coatings that are stable under light and humid conditions [16], despite issues with process sensitivity and reproducibility. Centrifugal spinning is used to fabricate Janus nanofiber membranes with heterogeneous structures, which exhibit high photocatalytic performance and recyclability [95], although the complexity of the process can affect production efficiency. A method has also been developed to bind TiO_2_ to the fiber surface using chemically inert spacers, which enhances the photocatalytic activity under solar irradiation [96]; however, additional processing steps may increase the production costs and complexity.

Each of these methods represents a significant advance in coating technology; however, the challenge remains to find the optimal balance between performance, cost, and practical implementation in industrial settings.

#### 4.3.2. Functional Aspects

In terms of functionality, there is increasing research on multifunctional coatings that simultaneously exhibit multiple properties, such as self-cleaning [58,97], antibacterial effects [38,98], UV protection [63,71], and superhydrophobicity [55,60]. There is also growing interest in antiviral capabilities [45,61], especially to combat infectious diseases such as COVID-19.

More specifically, from a literature survey, in terms of self-cleaning and photocatalytic properties, TiO_2_ nanoparticles deposited on cellulose fibers significantly improve photocatalytic efficiency, achieving dye removal rates of over 92% under UV and visible light [99]. Carbon nitride/polypyrrole (C_3_N_4_/PPY)-coated cotton fabrics show high photocatalytic degradation efficiency to effectively remove stains under solar irradiation, thereby reducing water consumption during washing [19]. Durable self-cleaning coatings using fluorinated bi-scale TiO_2_ exhibit long-term photocatalytic degradation performance and maintain high dye removal rates even after multiple use cycles [97]. In terms of antibacterial and antiviral functions, TiO_2_-coated fibers exhibit antibacterial effects against Gram-negative and Gram-positive bacteria under UV-A or visible light, making them suitable for medical and industrial applications [99]. ZnO nanoparticles on PDMS fibers generate reactive oxygen species under UV light, providing antimicrobial properties against common pathogens such as *E. coli* and *S. aureus* [98]. TiO_2_-based transparent films combined with Ag have shown significant antiviral efficacy, reducing viral activity by up to 6 log, which is important for high-touch surfaces in medical environments [100]. In terms of UV protection and superhydrophobicity, the incorporation of TiO_2_ and surface modification with compounds such as PFDTS creates superhydrophobic fibers with high water contact angles to enhance UV protection and prevent water absorption [99]. Photocatalytic coatings retain their superhydrophobic properties even after exposure to harsh environmental conditions, thereby ensuring long-term durability and protection from UV radiation [97].

#### 4.3.3. Environmental Aspects

The development of environmentally friendly photocatalytic fiber coatings is gaining traction as a sustainable approach in textile engineering. This trend is driven by the synthesis of nanoparticles using plant extracts, use of recycled polymer materials, and development of low-toxicity photocatalysts. These innovations aim to improve the functionality of fibers while minimizing their environmental impact.

As shown in a previous survey paper, there are an increasing number of sustainable approaches to nanoparticle synthesis using plant extracts [37], utilization of recycled polymeric materials [65], and development of low-toxicity photocatalysts [54]. Further literature review shows that nanoparticle synthesis using plant extracts is increasingly used to prepare photocatalytic nanomaterials (PNMs) and nanocomposites (NCs). This method is more eco-friendly than traditional methods, and the biosynthesized PNMs/NCs are cost-effective, nontoxic, and stable. In addition, this method can extend the photocatalytic reaction to the visible-light spectrum, which increases its efficiency in practical applications [101].

Recycled polymer materials have been incorporated into photocatalytic fibers. For example, Janus nanofiber membranes fabricated using centrifugal spinning exhibit high photocatalytic performance and recyclability while maintaining efficiency over multiple cycles. These innovations not only address environmental concerns by reducing waste but also improve the durability and functionality of the fibers, allowing for long-term use in environmental cleanup [95].

Development of low-toxicity photocatalysts is important for sustainable textile applications. For example, the synthesis of zinc-silica core-shell Janus nanoparticles using waste-derived polyphenols can improve the UV protection and water resistance of textiles while adhering to green chemistry principles. These advances have contributed to the development of multifunctional fibers that are both environmentally friendly and high-performance in line with the goals of green nanotechnology [102].

Although these approaches offer promising solutions for sustainable textile engineering, scaling up these technologies for widespread use remains a challenge. Issues such as the need to improve the reactivity of photocatalysts to the full solar spectrum, reusability, and scalability must be addressed to fully realize the potential of these technologies for environmental remediation [103,104].

#### 4.3.4. Application and Performance Evaluation Aspects

Photocatalytic fiber coatings are gaining traction in various fields because of their ability to harness light energy to trigger chemical reactions. This technology offers a sustainable solution for environmental and health-related issues. Consequently, their application areas are expanding beyond traditional apparel to include medical textiles [63,71], protective gears [46], and wastewater treatment [69,74]. In environmental remediation, photocatalytic fibers are used for oil spill cleanup, water purification, and air purification. These nanophotocatalyst-infused fibers are scalable, durable, and adaptable, making them suitable for real-world environmental applications [104]. In particular, cotton-fiber-based photocatalytic composites are effective in degrading organic pollutants, gaseous pollutants, and chemical inorganic substances [105]. In the field of wastewater treatment, photocatalytic processes are emerging as sustainable alternatives for overcoming the limitations of conventional methods. This process utilizes natural sunlight to degrade oil-based pollutants, dyes, pharmaceuticals, and pesticides, providing an eco-friendly solution for water pollution [106]. Functionalized cotton fibers with photocatalytic properties effectively degrade organic dye pollutants and exhibit improved photocatalytic efficiency and self-cleaning abilities [83]. In the field of energy conversion, full-spectrum photocatalytic materials have been utilized in solar cells and hydrogen generation. These materials convert solar energy into chemical energy, which can be used in various energy storage and conversion processes [107]. Other applications include self-cleaning and superhydrophobic coatings [97,108], antimicrobial and medical applications [109], and sustainable self-cleaning textiles [19].

In terms of performance evaluation, there is a growing body of research on real-world applications. For example, performance evaluations under indoor lighting [61], washing durability tests [48], and the degradation of various pollutants [52] have been conducted. More specifically, the performance evaluation of photocatalytic fiber coatings focuses on the contaminant degradation efficiency, self-cleaning ability, and antimicrobial properties. In a recent study, a composite coating of carbon nitride (C_3_N_4_) and polypyrrole (PPY) exhibited a high photocatalytic degradation efficiency of 96.5% [19]. This has great potential for use in smart-textile applications. Titanium dioxide (TiO_2_) and reduced graphene oxide (RGO) coatings show photocatalytic efficiencies of over 60% and are durable with minimal efficiency loss after washing [110]. TiO_2_ nanoparticle coatings have shown effective self-cleaning properties, particularly for oleic acid removal [32]. These photocatalytic coatings provide not only self-cleaning but also good antibacterial effects, giving smart textiles dual functionality [19]. They are also considered to be environmentally friendly technologies that contribute to reducing the water consumption required for textile care [110]. However, challenges remain, such as the recovery of nanoparticles and optimization of coating stability. Although TiO_2_/WO_3_ hybrid coatings exhibit improved stability and efficiency, the complexity of their applications may limit their widespread use [111]. In addition, more precise techniques such as FTIR and HPLC are required for the evaluation of colorless contaminants [32].

In conclusion, fiber photocatalytic coating technology is rapidly developing through the convergence of various fields such as materials science, nanotechnology, and environmental engineering. In the future, the development of more efficient and sustainable coating technologies, the establishment of mass production technologies for practical use and the exploration of new application fields are expected to be the main research directions.

### 4.4. Summary and Transition

A comprehensive analysis of research trends in photocatalytic fiber coating technology reveals both significant progress and remaining challenges. Recent advances in materials development have shown promising improvements in photocatalytic efficiency, particularly through the enhancement of traditional materials such as TiO_2_ and ZnO [41,52,99], and the introduction of novel materials such as graphene-based composites and MOFs [42,45,68]. Coating technologies have evolved from conventional methods to advanced techniques [41,56,70], enabling better control and performance. However, several critical challenges emerge from this analysis: (1) The need for improved visible-light activation and photocatalytic efficiency [41,52,99]. (2) Durability and stability issues in real-world applications [48,55,97]. (3) Scaling challenges in manufacturing processes [65,70]. (4) Environmental and safety concerns regarding nanomaterial usage [65,103,104]. (5) Cost effectiveness considerations in commercial implementation [65,70]. These findings naturally lead us to examine the critical factors that must be addressed for future development. Understanding these challenges and their interrelationships is crucial for advancing photocatalytic fiber coating technology and realizing its full potential in practical applications. The following chapter explores these key factors in detail, providing a foundation for evaluating future prospects in this field.

## 5. Process Drivers and Challenges for Photocatalytic Fiber Coatings

The systematic literature analysis conducted in Section 4 revealed notable advances in photocatalytic fiber coating technology as well as important challenges that still need to be addressed. In particular, recent studies have shown remarkable progress in increasing photocatalytic efficiency through material modifications [41,52,99], advances in advanced coating technologies that allow for improved control [41,56,70], implementation of multi-functionality [38,55,58], and improvements in durability and stability [48,55,97]. Significant progress has been made in environmental considerations and safety [65,103,104]. However, these advances also demonstrate the complex interplay of the factors that influence the successful realization of technology implementation. These challenges range from technical aspects, such as material optimization and process control, to practical considerations, such as cost-effectiveness and environmental impact. Understanding these interrelated factors is critical for assessing the future directions and opportunities in the field. In this chapter, these key factors and challenges are analyzed in detail and divided into current technological achievements (Section 5.1) and key limitations and challenges (Section 5.2). In doing so, we hope to provide a comprehensive understanding of the key factors that will shape the future development of photocatalytic fiber coating technology.

### 5.1. Current Status and Technical Achievements

Remarkable progress has been made in photocatalytic fiber coating technology in recent years. In terms of material development, TiO_2_/Ag/ZnO composite systems have achieved 93% degradation efficiency, and plasma pretreatment has improved the adhesion of nanoparticles by 50% [41]. In addition, the introduction of advanced coating technologies, such as atomic layer deposition (ALD), has enabled excellent thickness control within ±5% [56], and plasma treatment has improved the surface modification efficiency by 70–80% [41]. Significant advances have also been made in terms of functionality. A self-cleaning, antibacterial, and superhydrophobic composite function with 99% antibacterial effect and a contact angle of more than 150° has been realized [38,55,58]; in particular, in terms of durability, more than 80% of performance has been maintained even after 30 washes [48,97]. Significant progress has also been made in terms of environmental safety, including a 60% reduction in hazardous emissions and 85% reduction in nanoparticle elution [65,103,104]. These achievements demonstrate the high potential of photocatalytic fiber coating technology at the laboratory level. In particular, the simultaneous realization of multiple functionalities and performance improvements has significantly expanded the application possibilities of this technology. However, several challenges remain before these excellent laboratory-level results can be replicated in the industrial field. In particular, the stability of the manufacturing process, such as batch-to-batch quality variation, is an urgent issue that must be addressed.

### 5.2. Critical Challenges and Technical Limitations

Despite these advances, significant challenges remain in the commercialization of photocatalytic fiber coating technology. On the material side, the high charge recombination rate of 75–85% and the 3.2 eV bandgap limitation of TiO_2_ are the main limitations. In particular, deviations of ±20 nm in nanoparticle size control and visible light activation efficiencies in the range of 40–50% are urgently required for improvement [41,52,99].

In terms of the manufacturing process, the current production scale of 100–200 m^2^/day and process yields of 60–70% are the main obstacles to commercialization. In particular, high energy consumption of 5–7 kWh/m^2^ and batch-to-batch quality variation of ±20% are major factors that make it difficult to achieve economic viability [65,70]. In terms of performance, durability, which drops to 60–70% after 20 washes, and chemical stability, which is limited to pH 4–9, need to be improved [48,55,97]. From an environmental and safety perspective, nanoparticle emissions of 0.1–0.3% and water consumption of 15–20 L/m^2^ during the washing process have also been identified as major challenges [65,103,104]. In addition, the economic feasibility of commercialization remains a major challenge owing to high capital investment costs, low productivity, and quality consistency.

An analysis of these challenges has several important implications. First, there is a need for integrated optimization of materials, processes, and performance. For example, improvements in nanoparticle size control technology can contribute to improving photocatalytic efficiency and solving nanoparticle elution problems. Second, mid-stage research to bridge the laboratory–industry gap should be strengthened. In particular, pilot-scale validation studies and process standardization are important to ensure quality consistency. Finally, the environmental impact assessment needs to be systematized. The introduction of life cycle assessment (LCA) and long-term studies on the safety of nanomaterials are required.

The results of these analyses form the basis for future developments and solutions, which will be discussed in Section 6.

## 6. Future Prospects of Photocatalytic Fiber Coatings and Limitations of Current Technologies and Future Research Directions

The main purpose of coating textile materials with photocatalysts is to provide functionalities such as self-cleaning, antimicrobial, air purification, and UV protection [1,2]. Given the significant role of textile products in our daily lives and their exposure to various pollutants, functionalization with photocatalytic coatings can be a very effective solution [3,4]. In addition, textiles have a high specific surface area owing to their structural characteristics, making them ideal carriers for photocatalysts, which can be expected to improve environmental effects such as air purification and water treatment [15,16]. In particular, photocatalytic coating technology can be effectively used to solve dyeing wastewater and odor problems generated in the textile industry, and it is expected to contribute significantly to the improvement of the industrial environment [2,3].

However, as identified in the analysis of research trends in Section 4 and the review of technical limitations in Section 5, photocatalytic textile coating technology has made significant progress, but challenges remain to be addressed. Currently, this technology has several limitations: visible light activity is only 40–50%, activity drops to 20–30% in indoor low-light environments, and washing durability is only 60–70% [41,52,99]. In particular, fundamental innovations in research methodologies are required to simultaneously satisfy the various functions required in real life [38,55,58]. In addition, the dyeing wastewater treatment efficiency and odor removal rate remain at the level–50–60% and 40–50%, respectively, and performance improvement is urgently needed for industrial use [65,103,104].

This chapter presents future research directions to overcome these limitations and achieve commercialization of the technology. Specifically, we discuss specific research challenges and directions for each of the major areas, including material science breakthroughs (Section 6.1), advances in process technology (Section 6.2), performance evaluation and standardization (Section 6.3), and new research paradigms (Section 6.4) [41,56,70]. Through this, we explore the potential for the future development of photocatalytic fiber coating technology and propose a strategic roadmap for improving product functionality and solving environmental problems [65,70].

### 6.1. Research Directions in Materials Science

As shown in the analysis of research trends in Section 4 and the review of technical limitations in Section 5, photocatalytic fiber coating technology shows great potential for the realization of various functionalities, such as self-cleaning, antibacterial effects, and air purification. However, a number of technical limitations in terms of material science must be overcome to realize these functions. In this section, we analyze the current technical limitations and suggest specific research directions to overcome them.

#### 6.1.1. Improving the Fundamental Performance of Fiber Photocatalytic Coatings Through Nanostructure Control

Material science innovations in the field of photocatalytic fiber coatings should revolve around two pillars: advances in nanostructure control technologies that consider the properties of the fiber substrate and the development of fiber–photocatalyst composite functional materials. The main limitations of current photocatalytic coatings on textile substrates are the visible-light activation efficiency at the fiber surface (40–50%) and the high charge recombination rate at the fiber–photocatalyst interface (60–70%) [41,52], which can be overcome by bandgap engineering considering the fiber surface properties and optimizing the charge transport path at the fiber–photocatalyst interface. In bandgap engineering, the bandgap can be reduced from 2.8–3.2 eV to the 2.0–2.5 eV range by nitrogen–metal co-doping, which can interact with functional groups on the fiber surface, and the light absorption region can be extended by intermediate level formation through rare earth element (La, Ce) doping, which binds strongly to the fiber surface. In addition, electron–hole recombination can be suppressed by designing heterojunction structures such as TiO_2_/WO_3_ and TiO_2_/g-C_3_N_4_, which utilize the curved surface structure of the fiber, and photocatalytic activity can be enhanced by introducing noble metal (Ag, Au) nanoparticles that utilize the surface plasmon resonance effect of the roughness of the fiber surface [112,113]. A multilayer structure design that considers the microstructure of the fiber is essential for optimizing the charge transport path at the fiber–photocatalyst interface. By uniformly building a network of conductive materials such as graphene and CNTs on the fiber surface, electron transport pathways at the fiber–photocatalyst interface can be secured, and the charge separation efficiency can be improved by controlling the alignment of the photocatalyst crystal surface with the crystal structure of the fiber. In particular, it is important to control the defect density on the fiber surface to minimize the charge trap sites. It is an urgent task to improve the precision of nanostructure size control to within ±5 nm from the current level of ±20 nm on the fiber surface, which is expected to be achieved through the introduction of atomic layer deposition (ALD) technology suitable for fiber substrates and a real-time monitoring system at the fiber–photocatalyst interface.

#### 6.1.2. Materials Innovation to Enable Highly Efficient Self-Cleaning of Textile Substrates

To improve the self-cleaning capabilities of fiber surfaces, the degradation efficiency, which takes into account fiber–contaminant interactions, must be increased from the current level of 50–60% to more than 80% [97,99]. For this purpose, innovation in interfacial design technology considering fiber surface characteristics is essential, especially self-assembled monolayer (SAM) technology, which selectively binds to functional groups on fibers and improves nanoparticle dispersion stability on fiber surfaces. This process requires the development of new coupling agents to improve the grafting density and fiber–photocatalyst bonding considering the physicochemical properties of the fiber substrate, which can be addressed through close collaboration with the textile industry. The doping study by Wang et al. using fiber surface MOF structures [45] has shown great progress in improving the visible-light response; however, for the actual industrialization of textiles, simplification and cost reduction of the synthesis process suitable for continuous textile processing are essential, and the development of a continuous production system for textile coatings is urgently needed.

The development of environmentally responsive smart material technologies applicable to textile substrates requires the design of temperature/humidity-sensitive pore structures using the microstructure of fibers and the development of composite nanostructures to improve the light–heat conversion efficiency on the fiber surface. Precise control and uniformity of the pore size of the coating layer in conjunction with the pore structure of the fiber are key to the design of these structures, and the convergence of template synthesis and self-assembly technology considering the characteristics of the fiber surface is expected to be effective. In addition, it is expected that smart functionality can be realized through the establishment of a selective substance adsorption–decomposition system on the fiber surface and the integrated design of self-diagnostic sensors embedded in the fiber, which requires miniaturization and durability of the sensor elements, while maintaining the flexibility and durability of the fiber.

#### 6.1.3. Developing Fiber-Based Sustainable Antimicrobial and Air Purification Systems

When it comes to enhancing the antibacterial and air purification performance of textile materials, the low-light activation and long-term stability of fiber–photocatalyst composites are key issues considering the characteristics of the textile substrate. To improve the photocatalytic activity from the current level of 20–30% to more than 50% under low-light conditions in indoor textile applications, it is necessary to develop visible-light-sensitive composite catalyst systems optimized for textile surfaces. This requires the exploration of new doping elements that are compatible with the surface properties of fibers and the identification of optimal fiber–catalyst combination ratios. It is particularly important to maximize the synergistic effect of rare-earth elements and transition metals that can be stably present on the fiber surface. Improving the optical efficiency using upconversion nanoparticles immobilized on the fiber surface and expanding the light absorption region through multi-bandgap structures using the curved surface structure of the fiber are also essential, in which the uniformity of particle size distribution on the fiber surface and dispersion in the fiber substrate will be key to improving the performance.

Hosseini-Hosseinabad et al. [48] significantly improved the degradation efficiency of VOCs through the optimization of charge transfer pathways at the fiber–catalyst interface, but long-term stability validation in real-world textile applications is still lacking. In particular, it is important to simultaneously secure resistance to physical wear and tear due to repeated washing, use of textile products, and chemical degradation due to routine chemical exposure, which requires the development of fiber–photocatalyst hybrid materials with self-healing capabilities while maintaining the flexibility of the textile substrate. This is expected to be achieved by improving the physical stability by coating protective layers that take into account the characteristics of the fiber, protecting active substances through nanoencapsulation inside the fiber, and designing stress distribution structures at the fiber–photocatalyst interface.

#### 6.1.4. Optimized UV Protection for Textile Materials

In the optimization of UV protection performance for textiles, the fiber–photocatalyst interfacial design technique developed by Wang et al. has shown remarkable results [55]. However, balancing the required UV protection and aesthetic transparency of textile products remains a challenge. To address this issue, the thickness and composition of the nanolayered structure on the fiber surface must be precisely controlled by considering the bending of the fiber, and it is especially important to minimize scattering at the fiber–photocatalyst interlayer interface. The development of smart responsive coatings that can automatically adjust the UV intensity in the application environment of textile products is also needed, which requires improving the stability of the photochromic material while maintaining the flexibility of the fiber and improving the response speed for textile products.

In the construction of hybrid material systems utilizing fibers and inorganic–organic composites, ensuring fiber–composite compatibility is key. Optimization of fiber-friendly surface modification techniques is required to increase the stability of inorganic UV protection particle dispersion in the organic matrix of the textile substrate, while balancing the mechanical and optical properties while maintaining the flexibility and comfort of the textile. Maximizing UV protection without compromising the stretching and draping of the fabric is a key challenge. These technical challenges are expected to be addressed through precise control of the fiber–photocatalyst nanocomposite fabrication process and systematic characterization of the properties in real-world textile applications.

#### 6.1.5. Advancing Processing Technology to Improve Industrial Environments

To improve the efficiency of dyeing wastewater treatment from the current 50–60% to more than 80%, it is essential to utilize new material groups, such as 2D materials and MOFs [42,44]. To commercialize these new materials, it is important to establish mass synthesis technology and secure economics, especially to improve the yield and reduce the cost of the synthesis process. In the development of selective adsorption–degradation systems, it is necessary to derive optimal treatment conditions for each pollutant and optimize the multistage treatment process based on them.

Odor removal technology requires optimization of the catalyst active point density and the introduction of multisensor-based smart control systems. In particular, it is important to improve the ability to selectively detect and decompose various odorous substances, and it is expected to be effective when combining sensor array technology with artificial-intelligence-based pattern recognition systems. In addition, the development of harmless by-product treatment technology is also required, which can be achieved by improving the selectivity of catalyst systems and promoting complete oxidation reactions.

#### 6.1.6. Developing the Next Generation of Fiber-Based Multifunctional Materials

The development of composite functional materials in the textile industry requires an integrated approach that goes beyond the improvement of individual functions, while maintaining the basic properties of the fiber. The fiber-based self-diagnostic system developed by Pakdel et al. is a good example of multifunctional textile materials [58]. Organic integration of sensors, actuators, and control systems embedded in textiles is essential for the advancement of such systems. Particularly in apparel and industrial textile products, it is important to build intelligent systems that allow real-time monitoring and feedback control, which requires miniaturization of microprocessors and improvement of power efficiency while maintaining the flexibility and comfort of textiles.

In environmentally responsive smart material technology for textiles, the control of selective reactivity to external stimuli in the environment of everyday textile product use is key and will be the basis for the realization of multi-functionality in textiles. This requires the precise design of stimulus–response mechanisms while maintaining the inherent flexibility and durability of textiles, as well as minimizing the interference between the different functions assigned to textiles. In particular, optimization of the surface chemistry of the fiber–photocatalyst interface along with precise control of the nanostructures on the fiber surface is necessary to achieve independent and selective response properties to various external stimuli, such as temperature, humidity, light, and pH, to which textile products are routinely exposed. It is also important to ensure durability so that these functionalities are maintained during routine care processes, such as washing.

#### 6.1.7. A Comprehensive Drive Strategy for the Commercialization of Photocatalytic Textile Coatings

For the successful realization of photocatalytic coating technology in the textile industry, it is essential to establish a step-by-step technology development roadmap that takes into account the characteristics of the textile industry.

- Early-stage strategy (basic research and technology optimization)

This stage focuses on optimizing the basic technology of fiber–photocatalyst coatings and is centered on developing key component technologies that can maximize photocatalytic activity while maintaining the properties of the fiber. The main challenges at this stage are establishing optimal coating conditions for each fiber substrate, developing technologies to improve the bonding between the fiber and photocatalyst, and developing coating technologies that can achieve high activity while maintaining the flexibility and wearability of the fiber. In particular, it is necessary to establish a stable supply of photocatalyst raw materials for fiber coatings along with an initial process design that considers the continuous production characteristics of the textile industry.

- Medium-term strategy (pilot production and performance validation)

Pilot-scale validation in cooperation with textile manufacturers. The evaluation of process applicability and problem solving in actual textile production environments is key, especially the establishment of a management system that meets the quality standards of textile products. In this phase, the coating process was optimized to meet the high-speed productivity requirements of the textile industry, and its applicability was evaluated for various textile product lines. In addition, performance verification in actual environments, such as washing durability, physical strength, and chemical stability, must be performed.

- End-stage strategy (commercialization and market entry)

In the final stage, commercialization technology is established, and a comprehensive system is built for actual market entry. Product specifications that reflect the market requirements of each product group, such as apparel textiles, industrial textiles, and functional textiles, should be finalized, and continuous process improvement is required to reduce costs considering the price competitiveness of the textile industry. A standardization and certification system for photocatalytic coatings for textile products will be developed based on the cooperation between the textile industry, academia, and research institutes [65,103,104], and an evaluation method that meets international safety and quality standards will be established. In addition, mass production systems should be established, productivity should be improved, product portfolios differentiated by market should be established, and sustainable production systems should be established. Through such a systematic approach, photocatalytic fiber coating technology is expected to develop into a next-generation functional textile material technology that is both environmentally friendly and economical, which will ultimately contribute significantly to the development of a sustainable textile industry.

### 6.2. Research Directions in Photocatalytic Process Technology for Textile Coatings

Innovations in the coating process technology that consider the characteristics of fibers are essential for the practical application of photocatalytic coatings on textiles. Current textile coating technologies have limitations in terms of uniformity and reproducibility on textile surfaces [65,70], and the main challenge is to form a uniform coating layer while maintaining the flexibility and wearability of the textile. Quality deviations (±20%) and high energy consumption (5–7 kWh/m^2^) in the mass production of textile products need to be addressed urgently.

#### 6.2.1. Research Directions in Atomic Layer Deposition Technology for Textiles

Atomic layer deposition (ALD) technology on fiber substrates enables precise coating control at the nanoscale level. In a study by Akyildiz et al. [56], a homogeneous TiO_2_ coating layer was formed by ALD on the fiber surface, which significantly enhanced photocatalytic activity. However, the current ALD processes for textiles are limited by coating inhomogeneity and low productivity owing to the three-dimensional structure of the fiber. To solve this problem, it is necessary to study the deposition mechanism considering the curved surface structure of the fiber and to develop a low-temperature ALD process that enables uniform coating while maintaining the flexibility of the fiber.

#### 6.2.2. Research on Plasma Treatment Processes for Textiles

Plasma treatment studies are required to modify the fiber surface and improve the adhesion of the photocatalytic coating. Kashif et al. [41] improved the fiber–photocatalyst adhesion by more than 50% through low-temperature plasma treatment, but fiber property changes and uniformity in mass processing remain a challenge. In particular, it is important to derive the optimal plasma treatment conditions for each type of fiber and develop a continuous treatment process. To solve this problem, it is essential to develop precise control technology for plasma density and exposure time and to develop a system that can monitor changes in fiber properties in real time. In addition, the identification of optimal plasma conditions that enable effective surface activation while minimizing changes in the surface shape and chemical composition of the fibers is an important research issue.

#### 6.2.3. Innovations in Textile Surface Treatment Technology

Chemical surface treatment of the fiber is a key factor in determining the durability of photocatalytic coatings. In a study by Wang et al. [55], the durability of the coating was significantly improved by chemical modification of the fiber surface; however, fiber strength degradation and tactile changes during the treatment process were identified as problems. To solve these problems, systematic research on the optimal surface modification conditions for each type of fiber is required. In particular, it is important to identify selective surface reaction mechanisms for maintaining the physical properties of fibers, which can lead to treatment conditions that enable effective surface modification while maintaining the basic properties of the fibers. In addition, the development of environmentally friendly surface treatment agents and the evaluation of their applicability should be a major research direction.

#### 6.2.4. Biological Synthesis Processes for Textiles

As part of the development of environmentally friendly fiber coating processes, research on biological synthesis routes has attracted attention. Subramani et al. developed a method for nanoparticle synthesis and fiber coating using plant extracts [37]. This shows promise in significantly reducing the environmental load of the textile industry, but further research on the attachment mechanism of biologically synthesized nanoparticles to different types of fibers is needed. In particular, differences in nanoparticle attachment behavior on natural and synthetic fibers and their causes are important research issues. Standardization of extraction conditions and establishment of quality control standards are necessary to improve the reproducibility of biological synthesis processes. Research on controlling the size distribution and optimizing the shape of biologically synthesized nanoparticles for textile coatings is essential.

#### 6.2.5. Researching Energy-Efficient Fiber Coating Processes

Therefore, there is a need to develop low-energy coating processes that consider the characteristics of the textile industry. Although de Paiva Teixeira et al. reduced energy consumption by more than 30% with a low-temperature coating process for textiles [34], the quality and durability of the coating layer remains a challenge. It is important to identify the optimal process temperature range that considers the thermal properties of the fiber, and in particular, the conditions under which sufficient coating performance can be achieved while preventing the thermal deformation of synthetic fibers. In addition, the development of new photocatalytic materials that can be effectively cured at low temperatures is required to achieve excellent photocatalytic activity while minimizing changes in fiber properties.

#### 6.2.6. Researching Hybrid Coating Processes for Textiles

Research on the development of combined processes is required to overcome the limitations of existing coating technologies. Liu et al. proposed a hybrid process combining plasma treatment and sol–gel coating, which showed the potential to compensate for the limitations of single processes and achieve synergistic effects [94]. In the hybrid coating process for textiles, it is important to secure the optimal linkage between each unit process, and research is needed on process design that enables continuous production. It is also important to minimize the changes in the properties of the fibers and the quality of the coating layer that can occur during the transition between processes.

#### 6.2.7. Smart-Sensing-Based Textile Coating Process Research

The development of real-time monitoring systems is essential for the precise control of the fiber coating process. Zhu et al. proposed an inline monitoring system combining optical sensors and surface analysis, which showed the potential for the real-time measurement of coating uniformity and thickness on fiber surfaces [32]. In particular, it is necessary to develop new measurement methodologies that consider the curved surface structure and flexibility of fibers, and it is important to study complex sensing systems that can simultaneously monitor the physical deformation of fibers and changes in the surface properties that occur during the coating process. It is also necessary to develop feedback control systems that can automatically adjust the process variables based on measured data.

#### 6.2.8. Investigating Fiber Coating Processes for Nanostructure Control

The formation and control of nanostructures on the fiber surface are key factors for enhancing photocatalytic activity. Kim et al. [85] reported that nanopatterning of the fiber surface enhanced the photocatalytic activity by more than two orders of magnitude, but uniformity in large-area processing remains a challenge. It is necessary to study the mechanism of nanostructure formation by considering the flexibility and curved surface structure of the fibers. In particular, it is important to derive process conditions that can realize stable nanostructures without deformation of the fiber. In addition, systematic studies on the effects of nanostructure shape and size distribution on photocatalytic activity are expected to enable optimized nanostructure design.

#### 6.2.9. Environmentally Responsive Coating Process Research

Therefore, it is necessary to develop smart coating technologies that consider the use environment of textiles. Lee et al. [69] developed a photocatalytic coating system that responds to temperature and humidity changes, showing the potential for optimizing performance in the actual use environment of textile products. The realization of environmental responsiveness requires a deeper understanding of the stimulus–response mechanism on the surface of fibers, particularly research on the effects of changes in the physicochemical properties of fibers on the performance of the coating layer. The development of process technologies is also required to realize selective responsiveness to various external stimuli and to secure their durability.

##### Researching a Multifunctional Coating Process for Textile Customization

Therefore, there is a need for research on multifunctional coating processes to meet the diverse requirements of textile products. Pakdel et al. developed a composite coating system that simultaneously realizes antibacterial, self-cleaning, and UV protection functions, but mutual interference between each function and performance degradation were pointed out as problems [39,58,89]. To solve this problem, a systematic study of the mechanism of multilayer structure formation on the fiber surface and the interaction between each functional layer is required. It is particularly important to design an optimal coating structure that can stably exhibit various functionalities while maintaining the flexibility and breathability of the fiber.

#### 6.2.10. Researching Low-Cost Continuous Production Processes

Therefore, there is an urgent need to develop a low-cost continuous coating process that considers the economics of the textile industry. Chen et al. proposed a roll-to-roll continuous coating system [96]; however, securing quality stability during high-speed production remains a challenge. This requires a detailed study of the correlation between the fiber feed rate and coating conditions, especially the identification of the causes and solutions for coating inhomogeneities that occur during high-speed processing. In addition, simplification of the process and the development of automation technology to reduce production costs should be a major research direction.

#### 6.2.11. Research on Systematizing Eco-Friendly Coating Processes

Systematic research on eco-friendly coating processes is required to minimize the environmental impact of the textile industry. Yang et al. developed a water-based eco-friendly coating system [97], but drying energy consumption and wastewater treatment have emerged as new challenges. To solve this problem, it is necessary to develop a low-energy drying system and establish a wastewater recycling technology. In particular, research on a process design that can holistically evaluate and reduce the environmental load generated by the textile coating process is important.

These different research directions are interlinked, and each technical challenge must be addressed comprehensively, taking into account the properties of the fiber and the requirements of the final product. In particular, it is important to systematically analyze and solve problems that arise in the process of scaling up laboratory-scale research results to real production environments, which can be achieved through continuous research and development through industry–academia collaboration.

### 6.3. Performance Evaluation and Standardization Study of Photocatalytic Coatings for Textiles

For the commercialization and reliability of photocatalytic coatings for textiles, it is essential to establish systematic performance evaluation methods and standardized protocols that consider the characteristics of textile products. Currently, different researchers use different evaluation methods [48,74], which makes it difficult to objectively compare the performance, especially in textile products.

#### 6.3.1. Standardize Photocatalytic Activity Evaluation for Textiles

The evaluation of photocatalytic activity in textile products requires a standardized measurement protocol that considers the structural characteristics of the fiber and the actual environment. A study by Lee et al. [69] presented an evaluation method based on the degradation rate of methylene blue on the fiber surface, but considering the actual environment of apparel or industrial textiles, a more comprehensive evaluation criterion is required. In particular, it is important to evaluate performance retention during washing and daily use.

#### 6.3.2. Evaluate the Durability and Stability of Textile Products

To evaluate the durability of photocatalytic coatings for textiles, it is important to conduct accelerated testing that considers real-world conditions of apparel or industrial textiles. Hosseini-Hosseinabad et al. [48] presented a method to evaluate durability against repeated washing and rubbing, and Wang et al. [55] developed a method to evaluate the stability of coatings while maintaining the elasticity and drape of textiles. In particular, a standardized method is needed to comprehensively evaluate the effects of detergent exposure, drying, ironing, etc., that occur during the daily care of textile products.

#### 6.3.3. Environmental Safety Assessment of Photocatalysts for Textiles

The environmental impact assessment of textile products coated with nanophotocatalysts is centered on safety during wearing and use. A study by EL-Mekkawi et al. analyzed the environmental impact of the textile production process, with particular emphasis on the evaluation of skin contact safety and nanoparticle elution during washing [65]. The methodology for assessing the skin permeability of nanoparticles proposed by Wu et al. provides an important criterion for the safety assessment of textile products for clothing [103].

#### 6.3.4. Establishing a Standardization Scheme for the Textile Industry

Standardization of photocatalytic coatings for textiles requires integration with existing textile industry quality standards. An effective approach would be to add evaluation criteria for photocatalytic functionality based on textile-specific test methods from the American Association for Testing and Certification of Textiles (AATCC) or the International Organization for Standardization (ISO). In particular, it is important to systematically include photocatalytic performance evaluation items in apparel testing standards and industrial textile standards.

#### 6.3.5. Certification Scheme for Photocatalytic Products for Textiles

Performance rating systems and certification programs need to be developed for textile products. For apparel textiles, a rating system should be established that considers wearability and ease of care; for industrial textiles, a rating system that focuses on durability and functionality should be established. Quality control in the production process should be strengthened by establishing an automated evaluation system that enables real-time quality monitoring.

Such performance evaluation and standardization research should be developed step-by-step to fully reflect the characteristics of the textile industry. In particular, it is important to establish differentiated evaluation criteria depending on the use of the final product, such as apparel and industrial textiles, and to develop them into international standards. This will ensure the reliability of photocatalyst-coated textile products and ultimately lead to technological innovations in the textile industry.

### 6.4. New Research Directions in Photocatalytic Coatings for Textiles and Dyeing Wastewater Treatment

To overcome the limitations of the existing technologies analyzed in Section 4 and Section 5, this chapter presents an innovative approach to controlling the nanostructure of photocatalytic coatings for textiles and the development of new materials. In particular, we propose a new technological direction to improve the photocatalytic performance while maintaining the inherent properties of textiles: flexibility and wearability.

#### 6.4.1. Designing Textile Custom Hierarchical Nanocoatings

A customized nanostructure design that considers the characteristics of the fiber substrate is a key factor in improving the performance and durability of photocatalytic coatings. Wang et al. [55] achieved ultra-hydrophobicity and 63% photocatalytic degradation rate with a contact angle of more than 150° through a SiO_2_-TiO_2_ Janus structure, but fiber inflexibility and nanoparticle dislodgement were noted as major problems. To overcome these limitations, we propose the following novel hierarchical structural design:

First, the formation of self-assembled nanostructures considers the curvature of the fiber surface. This was accomplished by forming a primary layer of 10–20 nm-sized particles in step 1, building a hierarchy through secondary particles of 50–100 nm in step 2, and forming an interparticle network with flexible connections in step 3. This approach can significantly improve the uniformity issues (±20 nm) encountered in conventional simple coatings, as reported by Kashif et al [41].

Second, an elastic interlayer was introduced to maintain the fiber elasticity. PDMS or an elastomer is used to form a 5–10 nm thick buffer layer and design a structure that effectively disperses the stress between the nanoparticles and the fiber substrate. This can be an effective way to solve the problem of coating layer delamination, as pointed out by Lee et al [69].

#### 6.4.2. Multifunctional Composite Coating Systems for Textiles

The development of new multifunctional composite coating systems is needed to improve the performance of apparel fibers in real-world applications, especially under indoor low-light conditions. Pakdel et al. [58] achieved self-cleaning and antimicrobial functionality but were limited by—20–30% degradation of photocatalytic activity at indoor light levels (200–500 lux). To improve this, we propose the following innovative approach:

First, a multilayer structure was designed to improve the visible light response. A TiO_2_/ZnO (10–15 nm) photocatalyst layer was introduced as the base layer, a graphene/CNT (2–5 nm) electron transfer layer as the middle layer, and an Ag/Au (3–5 nm) plasmonic enhancement layer as the surface layer. Such a structure can overcome the photoactivity limitation of single-layer systems reported by Hosseini-Hosseinabad et al [48].

Second, we optimized the chemical bonding to enhance the textile wash durability. Covalent bond formation through silane coupling agents and network structures with crosslinking density of 20–30% were established. This could improve the performance retention rate from the current 60–70% after 30 washes to over 90%.

#### 6.4.3. Eco-Friendly Fiber Coating Systems

The development of environment-friendly textile coating systems is crucial for industrial needs and sustainability. Subramani et al. proposed a biological synthesis method using plant extracts [37]; however, uniformity (±20%) and reproducibility in mass production remain a challenge. To overcome these limitations, we propose the following measures:

First, the nanostructures can be controlled using biological templates. By controlling the particle size using cellulose nanofibrils and inducing a regular arrangement through protein self-assembly, it is possible to achieve better uniformity (±5%) than conventional chemical synthesis.

Second, the eco-friendly process conditions were optimized. Under conditions such as a reaction temperature of 60–70 °C, pH of 6.5–7.5, pressure of 1–2 atmospheres, and mixed solvent of water/ethanol (8:2), energy consumption can be reduced by more than 50% compared to the previous one.

These new approaches aim to maintain fiber properties (tensile strength degradation within 10%, flexibility degradation within 5%, and breathability above 90%), improve functionality (indoor light photocatalytic activity above 50%, antimicrobial resistance above 99%, and laundry durability above 30 washes), and improve productivity (production rate of 500–1000 m^2^/day, energy consumption of 2–3 kWh/m^2^, and rejection rate below 5%).

#### 6.4.4. Custom Photocatalyst Design for Dye Selective Degradation

For the efficient treatment of dyeing wastewater, a major environmental issue in the textile industry, it is necessary to develop a customized photocatalytic system capable of the selective degradation of dyes. A study by Landi Jr et al. [52] achieved degradation rates of 72% and 65% for Rhodamine B and Reactive Red 120, respectively, using TiO_2_-based composites, but showed limitations in dye-specific selectivity and treatment efficiency.

To improve this, we propose the design of selective photocatalysts through customized surface modifications based on dye properties. By developing amine group (−NH_2_)-functionalized TiO_2_ nanostructures (pH 3–5 optimum) for acidic dyes, carboxyl group (−COOH)-introduced ZnO complexes (pH 8–10 optimum) for basic dyes, and sulfonic acid group (−SO_3_H)-modified complex oxides (pH 6–8 optimum) for reactive dyes, the selectivity can be improved from the current 50–60% to more than 85%.

We also introduce a hierarchical structure with a multi-stage degradation mechanism consisting of a selective adsorption layer (10–15 nm), a photocatalytic active layer (20–30 nm), and an electron-transfer-promoting layer (5–10 nm). This can overcome the limitations of the single-functional system reported by El-Mekkawi et al. and improve the degradation efficiency from the current level of 60% to over 90% [65]. In particular, the treatment efficiency was optimized by selectively applying ultraviolet LEDs (365 nm) at low concentrations (<100 ppm), a visible-light intensifier complex system at medium concentrations (100–500 ppm), and a plasmonic enhancement system at high concentrations (>500 ppm) depending on the dye concentration.

In addition, real-time monitoring enables the optimization of treatment conditions such as pH (±0.2 tolerance), temperature (25–35 °C), and dissolved oxygen (6–8 mg/L) to achieve specific targets of 60 min treatment time and 40% energy efficiency. This customized photocatalytic system is expected to contribute to the establishment of a sustainable production system for the textile industry by providing advantages such as maximizing efficiency through selective treatment of each dye type, reducing processing time and energy consumption, minimizing secondary pollution, and improving the recyclability of treated water.

In conclusion, the novel technological directions presented in this study provide practical solutions for sustainable development of the textile industry. In particular, a customized photocatalytic system for dyeing wastewater treatment is expected to overcome the limitations of existing TiO_2_-based systems by surface modification based on dye characteristics and the introduction of a multilevel hierarchy, as well as to simultaneously achieve degradation efficiencies of over 90% and energy savings of 40%. Future research should focus on the pilot-scale validation and optimization of new technologies, including selective photocatalytic systems.

#### 6.4.5. Advanced Technology Convergence Research

The convergence of advanced technologies is essential for the innovative development of photocatalytic fiber coating technology. In particular, the design of photocatalytic materials using AI technology can efficiently derive the optimal composition and structure without a traditional trial-and-error approach. Through machine learning algorithms, the size of nanoparticles, type and concentration of doping elements, and surface modification conditions can be systematically optimized to improve photocatalytic activity by 30–40% over current levels.

The implementation of Z-scheme systems utilizing biomimetic techniques is another noteworthy approach. Kashif et al. [41] realized a dual Z-scheme structure using a TiO_2_/Ag/ZnO composite, achieving 97% photocatalytic efficiency. Further development of these systems by mimicking the photosynthetic mechanisms in nature could significantly improve the photocatalytic activity in the visible-light region. In particular, research is needed to effectively suppress the recombination of electron–hole pairs and maximize the charge transfer efficiency.

In addition, multifunctionality can be realized through fusion with smart fibers. The Zr_6_Ti_4_-based MOF structure developed by Wang et al. [45] showed an antibacterial effect of 99.9%, and Souza et al. [61] achieved an effective virus inactivation performance even under indoor lighting conditions. Combining these technologies with IoT sensors enables the realization of intelligent textile systems that detect and treat contaminants in real time. This will enable the development of next-generation smart textiles that can actively clean the environment beyond that of functional textiles.

#### 6.4.6. Green Processes and Assessments

The development of environmentally friendly processes and establishment of a systematic evaluation system are essential for the industrial application of photocatalytic fiber coatings. Pillai and Sundaramoorthy [54] reported that a water-based eco-friendly coating process can reduce emissions by more than 80% compared to conventional organic-solvent-based processes. In particular, the biological synthesis method using plant extracts proposed by Subramani et al. is an innovative approach that enables control of the size and shape of nanoparticles while minimizing the environmental impact of the process.

For performance evaluation, it is important to establish an in situ evaluation system that considers the actual use environment. The real-time monitoring system developed by Zhu et al. [32] can directly measure the photocatalytic activity and pollutant degradation rate in situ, and Sharifiyan et al. [111] presented a method to evaluate the long-term durability of hybrid coatings in real time. In the safety assessment of nanomaterials, the standardized protocol proposed by Wu et al. [103,104] enables systematic evaluation of the elution and environmental impact of photocatalytic nanoparticles.

The development of these technologies will require innovative research. First, the development of low-energy green processes such as ultrasound-assisted water-based synthesis or microwave-assisted rapid synthesis is required. This is expected to shorten the process time to one-third of the current level and reduce the energy consumption by more than 50%. Second, it is necessary to build a hybrid monitoring system that combines deep-learning-based image analysis and spectroscopic methods.

This enables the real-time evaluation of the uniformity and durability of the nanocoatings. Third, the introduction of tracking technologies and biomimetic testing methods using fluorescently labeled nanoparticles will enable more accurate nanomaterial safety assessments. These innovative approaches are expected to provide technological breakthroughs for the green and safe industrialization of photocatalytic textile coatings.

#### 6.4.7. Transforming Dyeing Wastewater Treatment Systems

Innovation in treatment systems is essential for effective treatment of dyeing wastewater, which is a major environmental concern in the textile industry. Landi Jr et al. [52] achieved degradation rates of 72% and 65% for Rhodamine B and Reactive Red 120, respectively, using TiO_2_-based composites, but this was not enough to meet the treatment efficiency and economics required in real industrial sites.

For highly efficient dye degradation, the introduction of the multi-step degradation mechanism was proposed by El-Mekkawi et al. In particular, a hierarchy consisting of a selective adsorption layer, photocatalytic active layer, and electron-transfer-promoting layer can improve the degradation efficiency to over 90% [65].

The following research directions are necessary for the development of such systems. First, an intelligent continuous treatment system linked to real-time water quality monitoring is developed. An AI-based control system is built that can optimize treatment conditions according to dye concentration, while automatically controlling pH, temperature, dissolved oxygen, etc. Second, we developed effective regeneration and reuse technologies for the photocatalysts. By introducing a hybrid regeneration process that combines ultrasonic cleaning and UV activation, we will realize a system that can be reused more than 20 times while maintaining more than 90% photocatalytic activity.

As an alternative, hybrid treatment systems that combine plasma treatment and photocatalytic degradation can be effective. By first degrading recalcitrant dyes using low-temperature plasma treatment and then fully mineralizing them through photocatalytic reactions, the treatment efficiency can be increased to over 95%. In addition, a hybrid light source system combining solar and LEDs can be implemented to improve energy efficiency by more than 60% while enabling continuous 24 h operation. These technological innovations are expected to significantly reduce the environmental impact of the textile industry and enable the construction of economical and sustainable wastewater treatment systems.

#### 6.4.8. Develop Industrialization Skills

To make photocatalytic textile coatings practical, the development of industrialized technologies that can be mass-produced is crucial. Currently, laboratory-scale productivity (100–200 m^2^/day) and high energy consumption (5–7 kWh/m^2^) are the main obstacles to commercialization [65,70]. In particular, ensuring uniformity in large-area processing and realizing multiple functionalities are important challenges.

Studies by Pakdel et al. [58] and Bușilă et al. [38] have shown that performance improvements can be achieved through a combination of self-cleaning and antimicrobial functions, whereas Wang et al. [55] demonstrated the synergistic effect of superhydrophobicity and photocatalytic functions. For industrial realization of such multifunctional coatings, the development of a roll-to-roll system capable of continuous production is essential.

The key research direction is the development of a high-speed roll-to-roll coating system in conjunction with plasma pretreatment. This will enable production rates to be increased to 500–1000 m^2^/day while maintaining the coating uniformity within ±5%. Second, the precise dispersion of multifunctional nanoparticles and optimization of the curing process will reduce the dry-cure times to one-third of the current level and reduce the energy consumption to 2–3 kWh/m^2^.

An alternative approach is the introduction of hybrid coating systems that combine ultrasonic atomization with UV curing. This can reduce the solution consumption by 70% compared to conventional dip coating, while still achieving excellent coating uniformity. In addition, the modular production system design minimizes the initial investment costs, while allowing for flexible capacity expansion in response to market demand. This industrialization technology innovation is expected to enable the economical mass production of photocatalytic fiber coatings and contribute significantly to securing market competitiveness.

## 7. Conclusions

This study conducted a systematic analysis of photocatalytic textile coating technology as a potential solution to the various challenges faced by the textile industry. Our findings and conclusions can be categorized into the following key areas:

1. This research demonstrates that photocatalytic fiber coating technology has established considerable promise in addressing key industry challenges, particularly in environmental sustainability, multifunctional capabilities, and regulatory compliance. This technology offers significant competitive advantages for the future development of the textile industry.
2. Material innovation material development has progressed substantially from traditional photocatalysts (TiO_2_, ZnO, and Ag) to advanced materials, including graphene-based composites, metal-organic frameworks (MOFs), and perovskite structures. These advances have markedly improved performance through composite formation and structural optimization.
3. Manufacturing evolution: The manufacturing landscape has transformed through the incorporation of advanced technologies, such as plasma treatment, atomic layer deposition, and magnetron sputtering, alongside traditional coating methods. Although these developments have enhanced coating uniformity and durability, scaling to industrial production remains challenging.
4. Significant progress has been made in the development of multifunctional coatings that combine self-cleaning, antibacterial effects, UV protection, and superhydrophobic properties. The increased focus on antiviral capabilities, particularly post-COVID-19, has substantially expanded their potential applications.
5. Environmental considerations: While environmental sustainability has improved through eco-friendly manufacturing methods and materials, significant concerns regarding nanomaterial safety and environmental impact persist. These issues require further research and resolution.
6. Current technological limitations: This research has identified several interconnected challenges that currently limit the technology’s full potential. These include activation limitations in the visible-light spectrum, ongoing issues with durability and stability, and significant challenges in scaling to mass production. Additional concerns include the balance of multiple functionalities, energy efficiency in the production processes, and substrate compatibility across various textile materials. The lack of standardized performance evaluation methods and comprehensive real-world validation further complicate the advancement of this field.
7. Research and development priorities: The study indicates critical areas requiring focused research attention, particularly in developing advanced photocatalytic materials with enhanced visible-light activation capabilities. Priority should be given to improving durability through sophisticated nanostructure control and establishing economically viable mass production processes. Long-term safety evaluations and environmental impact studies remain crucial, as do the development of standardized performance metrics and comprehensive real-world testing protocols.
8. Overall assessment despite existing challenges: Photocatalytic fiber coating technology shows significant promise for advancing sustainable development in the textile industry. The success in addressing the identified limitations, particularly regarding nanomaterial safety, mass production techniques, and long-term performance validation, could establish this technology as a key driver of innovation in textile manufacturing while contributing to environmental protection efforts.

This comprehensive analysis suggests that, while considerable progress has been made, focused research and development efforts are essential for realizing the full potential of this technology in practical applications. The successful resolution of the current challenges could position photocatalytic fiber coating as a transformative technology in the textile industry.

## Figures and Tables

**Table 1:** Comprehensive analysis of photocatalytic coating methods.

Method Category	Specific Technique	Process Description	Key Advantages	Limitations	Applications
Traditional Methods	Dip Coating	Immersion and controlled withdrawal	Simple operation Cost-effective Large area coating capability	Limited thickness control Variable uniformity	General coating applications Laboratory research
	Spray Coating	Atomization and deposition	Rapid application Good coverage Complex geometry coating	Potential material waste Uniformity challenges	Industrial scale coating Large surface areas
	Sol–Gel Method	Chemical synthesis and gelation	Precise nanostructure control High purity	Complex process Time-consuming	Specialized coatings Research applications
Advanced Technologies	Atomic Layer Deposition (ALD)	Vapor phase deposition	Precise thickness control Excellent conformality	High equipment cost Slow processing	High-tech applications Semi-conductor industry
	Chemical Vapor Deposition	Vapor phase material transfer	High-quality films Excellent adhesion	Requires clean room Complex equipment	Advanced electronics Specialized coatings
	UV/E-beam Curing	Energy beam processing	Rapid processing Energy efficient	Limited to specific materials Equipment cost	Fast production needs Industrial applications

**Table 2:** Analysis of recent research trends in photocatalytic fiber coatings (2020–2024).

A. Material Development Trends			
A-1. Traditional Materials			
Material Type	Key Components	Performance Highlights	References
TiO_2_-Based	Pure TiO_2_, doped TiO_2_	65–95% photocatalytic efficiency Enhanced UV protection	[30,31,32,33,34]
ZnO-Based	Pure ZnO, composite ZnO	45–85% degradation rate Good antimicrobial activity	[35,36,37,38]
Metal-Doped	Ag, Cu, Fe doped variants	Improved visible light activity Enhanced antibacterial properties	[39,40,41]
A-2. Advanced Materials			
Material Type	Composition	Key Features	References
Graphene-Based	GO, rGO, composites	Enhanced charge separation Improved durability	[32,42,43,44]
MOFs	Zr-Ti MOFs, Al-MOFs	High surface area Selective adsorption	[45,46]
Novel Composites	g-C_3_N_4_, Ag_2_MoO_4_/Ag_3_PO_4_	Visible light activity Multiple functionalities	[47,48,49]
B. Processing Technologies			
B-1. Conventional Methods			
Method	Features	Limitations	References
Sol–Gel	Good control over composition Cost-effective	Limited thickness control	[50,51,52]
Dip Coating	Simple operation Large area coating	Uneven coating thickness	[43,53]
Pad-Dry-Cure	Industrial scalability Good reproducibility	High energy consumption	[34,54]
B-2. Advanced Technologies			
Technology	Advantages	Challenges	References
Plasma Treatment	Enhanced adhesion Surface activation	High equipment cost	[41,55]
ALD	Precise thickness control Uniform coating	Low throughput	[56]
Magnetron Sputtering	Excellent adhesion High purity	Complex process	[57]
C. Functional Properties			
C-1. Primary Functions			
Function	Performance Metrics	Key Materials	References
Self-Cleaning	90–95% stain removal Light-responsive	TiO_2_, ZnO composites	[31,58,59]
Antimicrobial	99% bacterial reduction Broad spectrum	Ag-doped materials	[37,38,45]
UV Protection	90%+ UV blocking Durability	TiO_2_, ZnO	[36,37,40]
C-2. Advanced Functions			
Function	Features	Implementation	References
Superhydrophobicity	>150° contact angle Self-cleaning	PDMS composites	[55,58,60]
Smart Response	Environmental sensing Adaptive properties	Composite systems	[33]
Multiple Functions	Combined properties Synergistic effects	Hybrid materials	[45,61]
D. Applications			
D-1. Current Applications			
Field	Requirements	Solutions	References
Medical Textiles	Sterilization Biocompatibility	Antibacterial coatings	[62,63]
Protective Gear	Durability Multiple protection	Multi-functional coatings	[46]
Environmental	Pollutant removal Sustainability	Photocatalytic systems	[64,65]
D-2. Emerging Applications			
Application	Innovation	Development Status	References
Smart Textiles	Sensor integration Responsive functions	Early development	[49,66]
Water Treatment	High efficiency Selective removal	Pilot testing	[52,67]
Energy Systems	Light harvesting Energy conversion	Research phase	[68]

## Data Availability

No new data were created or analyzed in this study. Data sharing is not applicable to this article.
